# Limiting protease production plays a key role in the pathogenesis of the divergent clinical isolates of *Staphylococcus aureus* LAC and UAMS-1

**DOI:** 10.1080/21505594.2021.1879550

**Published:** 2021-02-04

**Authors:** Joseph S. Rom, Karen E. Beenken, Aura M. Ramirez, Christopher M. Walker, Ethan J. Echols, Mark S. Smeltzer

**Affiliations:** a Department of Microbiology and Immunology, University of Arkansas for Medical Sciences, Little Rock, Arkansas, USA; b Department of Orthopaedic Surgery, University of Arkansas for Medical Sciences, Little Rock, Arkansas, USA; c Department of Pathology, University of Arkansas for Medical Sciences, Little Rock, Arkansas, USA

**Keywords:** *Staphylococcus aureus*, UAMS-1, USA200, *codY*, *rot*, *sarA*, *sigB*, sepsis, biofilm, protease

## Abstract

Using the USA300, methicillin-resistant *Staphylococcus aureus* strain LAC, we previously examined the impact of regulatory mutations implicated in biofilm formation on protease production and virulence in a murine sepsis model. Here we examined the impact of these mutations in the USA200, methicillin-sensitive strain UAMS-1. Mutation of *agr, mgrA, rot, sarA* and *sigB* attenuated the virulence of UAMS-1. A common characteristic of *codY, rot, sigB*, and *sarA* mutants was increased protease production, with mutation of *rot* having the least impact followed by mutation of *codY, sigB* and *sarA*, respectively. Protein A was undetectable in conditioned medium from all four mutants, while extracellular nuclease was only present in the proteolytically cleaved NucA form. The abundance of high molecular weight proteins was reduced in all four mutants. Biofilm formation was reduced in *codY, sarA* and *sigB* mutants, but not in the *rot* mutant. Eliminating protease production partially reversed these phenotypes and enhanced biofilm formation. This was also true in LAC *codY, rot, sarA* and *sigB* mutants. Eliminating protease production enhanced the virulence of LAC and UAMS-1 *sarA, sigB* and *rot* mutants in a murine sepsis model but did not significantly impact the virulence of the *codY* mutant in either strain. Nevertheless, these results demonstrate that repressing protease production plays an important role in defining critical phenotypes in diverse clinical isolates of *S. aureus* and that Rot, SigB and SarA play critical roles in this regard.

## Introduction

*Staphylococcus aureus* is among the most prominent of bacterial pathogens and is a serious public health threat. The prominence of *S. aureus* as a pathogen can be attributed to its ability to produce a diverse array of virulence factors in a carefully orchestrated fashion mediated through a complex and highly interactive regulatory circuit [1,2]. The combination of its arsenal of virulence factors, and the ability to precisely control their production to meet the demands of different microenvironments within the human host, allow *S. aureus* to cause a diverse array of infections ranging from skin and soft tissue infections to sepsis and disseminated secondary infections of essentially any tissue [3–5]. The treatment of these infections is increasingly compromised by the persistent emergence of *S. aureus* strains that are resistant to front-line antibiotics, most notably oxacillin [6]. Indeed, the Infectious Disease Society of America (IDSA) has recognized *S. aureus* as one of the ESKAPE pathogens [7] owing to its prominence as a cause of human infection and the decreasing availability of antibiotics to treat these infections. Additionally, *S. aureus* has the capacity to form a robust biofilm, which at least in part accounts for its prominence as a cause of osteomyelitis and infections associated with indwelling medical devices [8]. These biofilms confer a therapeutically relevant level of intrinsic resistance to antibiotic therapy and host defenses, thus necessitating an interdisciplinary clinical approach that most often includes surgical debridement augmented by some form of local antibiotic delivery, and even then, recurrent infections and even amputation are an all-too-common occurrence [9,10].

These factors have created an urgent need for alternative strategies to combat *S. aureus* infections. Although a considerable effort has been put forth to develop an effective vaccine, thus far, these efforts have failed and at present, no such vaccine exists [11]. Therapies based on the use of bacteriophage and/or phage-associated lytic proteins have recently garnered attention but have inherent limitations with respect to strain specificity and have not yet achieved widespread clinical use [12]. Nanotechnology is also being explored as a means of combatting infectious disease. For example, in the specific context of *S. aureus*, we examined laser irradiation of antibody-conjugated gold nanoparticles, including gold nanocages containing daptomycin, to achieve immediate photothermal killing and controlled antibiotic release directly at the bacterial cell surface [13–17]. Given the arsenal of *S. aureus* virulence factors, another approach has been to target key elements within the regulatory circuit controlling the production of these virulence factors [18,19]. The two best-studied regulatory loci in this regard are the accessory gene regulator (*agr*) and the staphylococcal accessory regulatory (*sarA*), and putative inhibitors of the function of these loci have been described [20–22].

Based on our specific interest in the clinical problem of biofilm-associated orthopedic infections, we initially focused our efforts on identifying regulatory loci that are required for biofilm formation. This led us to focus on *sarA*, mutation of which limits biofilm formation in diverse clinical isolates of *S. aureus* [23]. We subsequently evaluated an array of *S. aureus* regulatory mutants based on their capacity to form a biofilm, and the results confirmed that mutation of *sarA* limits biofilm formation to a greater extent than mutation of any other regulatory locus we examined [24]. Moreover, in regulatory mutants that exhibited an enhanced capacity to form a biofilm, mutation of *sarA* reversed this effect [24,25]. Given the diversity of infections caused by *S. aureus*, we then extended these studies to examine the impact of *sarA* and other regulatory loci found to impact biofilm formation on pathogenesis in a murine sepsis model [26]. These studies demonstrated that mutation of *codY, sarA*, and *sigB* in the USA300 strain LAC limited virulence, and phenotypic characterization of these mutants suggested that this may be due to the increased production of extracellular proteases and a corresponding decrease in the accumulation of high molecular weight extracellular proteins.

Our previous sepsis studies were done with LAC because it is a prominent cause of serious community-acquired infections [27]. However, yet another factor that complicates the development of alternative therapies to combat *S. aureus* infections is the genetic and phenotypic diversity among clinical isolates. Given our interest in orthopedic infections, we focused much of our work on an isolate obtained directly from the bone of an osteomyelitis patient during surgical debridement [28]. This isolate, which was designated UAMS-1, is a USA200, methicillin-sensitive strain that is very distinct by comparison to USA300 isolates like LAC [29,30]. At a genetic level, examples of potentially relevant differences include *cna*, which encodes the primary collagen-binding adhesin produced by *S. aureus* [31] and is present in in UAMS-1 but not in LAC and, conversely, the absence of the genes encoding the Panton-Valentine leucocidin (PVL) and the regulatory loci *sarT* and *sarU*, all of which are present in LAC [29]. Phenotypically, UAMS-1 is characterized by its ability to form a robust biofilm and limited production of extracellular toxins, while LAC is highly toxigenic owing to the increased expression of *agr* [27]. LAC also produces alpha toxin, while UAMS-1 does not owing to a point mutation in the corresponding gene (*hla*) [32]. Any alternative therapeutic strategy targeting *S. aureus* specifically would be most effective if it were not limited by such differences. For this reason, we extended our previous experiments that were limited to LAC to assess the impact of these same regulatory mutations in UAMS-1.

## Materials and methods

### Bacterial strains and growth conditions

The bacterial strains used in these experiments are listed in Tables 1 and 2. Relevant citations are included for UAMS-1 and LAC regulatory mutants described in previous reports [24,26,28,33,34]. The only exception was the UAMS-1 *fur* mutant, which was generated by phage-mediated transduction from the existing mutant in the Nebraska Transposon Mutant Library (NTML) into LAC and then into UAMS-1 [35]. A LAC *sarA* mutant unable to produce aureolysin, ScpA, SspA, SspB, and any of the six *spl*-encoded proteases (SplA-F) was generated as previously described [36]. The protease-deficient LAC *codY, rot*, and *sigB* mutants were generated by phage-mediated transduction of each regulatory mutation into this protease-deficient strain.

**Table 1.** LAC *S. aureus* strains used in this study

Strain	Genotype	References
UAMS-2279^a^	Wild type	36
UAMS-4446	*spa::erm*	26
UAMS-3001	∆*aur*/∆*scpA*/∆*sspAB*/*spl::erm*	36
UAMS-2294	*sarA::kan*/*neo*	36
UAMS-3002	*sarA::kan*/*neo*/∆*aur*/∆*scpA*/∆*sspAB*/*spl::erm*	36
UAMS-3079	*agr::tet*/*min*	26
UAMS-4177	*codY::erm*	24
UAMS-4571	*codY::spec*/∆*aur*/∆*scpA*/∆*sspAB*/*spl::erm*	This work
UAMS-4244	*fur::erm*	24
UAMS-4198	*mgrA::cat*	24
UAMS-4291	*rot::spec*	24
UAMS-4585	*rot::tet*/∆*aur*/∆*scpA*/∆*sspAB*/*spl::erm*	This work
UAMS-4204	*sigB::erm*	24
UAMS-4522	*sigB::tet*/∆*aur*/∆*scpA*/∆*sspAB*/*spl::erm*	This work

*^a^*Variant of the clinical isolate LAC which has been cured of the erythromycin resistance plasmid as previously described (36].

**Table 2.** UAMS-1 *S. aureus* strains used in this study

Strain	Genotype	References
UAMS-1	Wild type	28
UAMS-1471	∆*nuc*	33
UAMS-4738	∆*aur*/*scpA::tet*/∆*sspAB*/∆*spl*	This work
UAMS-929	*sarA::kan/neo*	34
UAMS-4739	*sarA::kan*/*neo*/∆*aur*/*scpA::tet*/∆*sspAB*/∆*spl*	This work
UAMS-155	*agr::tet*	34
UAMS-1624	*codY::erm*	24
UAMS-4740	*codY::erm*/∆*aur*/*scpA::tet*/∆*sspAB*/∆*spl*	This work
UAMS-4703	*fur::erm*	This study
UAMS-4185	*mgrA::cat*	24
UAMS-4293	*rot::tet*/*min*	24
UAMS-4750	∆*rot*/∆*aur*/*scpA::tet*/∆*sspAB*/∆*spl*	This work
UAMS-1274	*sigB::tet*	24
UAMS-4742	∆*sigB*/∆*aur*/*scpA::tet*/∆*sspAB*/∆*spl*	This work

A derivative of UAMS-1 unable to produce aureolysin or SspA/SspB was generated as previously described [37]. To generate a derivative of this strain unable to produce ScpA, the resistance marker present in the *scpA* mutant present in the NTML was switched from erythromycin to tetracycline using previously described methods [38]. This mutation was then transduced into the UAMS-1 mutant unable to produce aureolysin and SspA/SspB. Unlike LAC, which encodes the entire six-gene *splA-F* operon, UAMS-1 encodes a truncated version limited to *splC-F* (data not shown). To generate a UAMS-1 *splC-F* mutant, allelic exchange was performed using the protocol previously described [38]. Specifically, primer sets (Table 3) were used to amplify a 722 bp fragment and a 544 bp fragment upstream and downstream of the *spl* operon, respectively. These fragments were digested with SacII, ligated together, and PCR amplified using the upstream primer used to amplify the 722 bp fragment together with the downstream primer used to amplify the 544 bp fragment primer sets were used to amplify flanking regions of the UAMS-1 *spl* gene cluster to generate 722 bp and 544 bp fragments, respectively. These fragments were digested with SacII, ligated together, and PCR amplified to generate a construct containing the regions upstream and downstream of the *spl* operon but lacking the operon itself. The PCR product was then digested with EcoRI, cloned into the temperature-sensitive vector pJB38, and transformed into TOP10 *E. coli* (Invitrogen, Cat. # C404010) [38]. Once confirmed, the plasmid was isolated from *E. coli* and transformed into *S. aureus* strain RN4220 before being transduced into the derivative of UAMS-1 unable to produce aureolysin, SspA, SspB and ScpA. To induce recombination, the recipient strain was incubated at the non-permissive temperature (42°C) overnight with 10 μg ml^−1^ chloramphenicol to induce a single crossover event. Bacteria were then sub-cultured and grown at 30°C without antibiotic selection to induce plasmid loss. Bacteria were then recovered on tryptic soy agar (TSA) containing 1 μg ml-1 anhydrotetracycline for counter-selection, thus resulting in a UAMS-1 mutant unable to produce any of the 10 primary extracellular proteases. This mutant was used as a recipient strain to transduce regulatory mutations into this derivative of UAMS-1 that was unable to produce any extracellular protease.

**Table 3.** Primers used for constructing mutants

Name	Sequence (5ʹ → 3ʹ)
*spl*-UP-For	CCGAATTCCATAGTTAGAACAATGAATTAG
*spl*-UP-Rev	GGACCTCCGCGGCTCCTTTACAATTTGAGATA
*spl*-DOWN-For	GGACCTCCGCGGTAAGAAATTCATTGCAGATA
*spl*-DOWN-Rev	CCGTCGACTTTCAGATAAGAAGCAATAG
*rot*-UP-attB1-For	GGGGACAAGTTTGTACAAAAAAGCAGGCTGTATTAGGGGTAGGACTTGGCGGTTTG
*rot*-UP-Rev	ATGACTGGATCCCTACAAGTGTAAATAAACTTGCTTTC
*sigB*-UP-attB1-For	GGGGACAAGTTTGTACAAAAAAGCAGGCTCGGATGGTGTGACTGAAGCTAGAAATAGTG
*sigB*-UP-Rev	ATGACTGGATCCCCATTGGTTAATTTGCTCAGGTGAAACTTC
*rot*-DOWN-For	ATGACTGGATCCGATTGCAAGTAGAGCAACAGCAATGC
*rot*-DOWN-attB2-Rev	GGGGACCACTTTGTACAAGAAAGCTGGGTGAAGACTATGACATGATGATGGCTACTGCC
*sigB*-DOWN-For	ATGACTGGATCCCGGCAATTAAGAAATTACAAGAAGCAGCAC
*sigB*-DOWN-attB2-Rev	GGGGACCACTTTGTACAAGAAAGCTGGGTTTAGATGTAGTTAACACACCTTGATGATAC

Existing *sarA* and *codY* mutants [24,34] were used as transduction donors to move these regulatory mutations into this protease-deficient derivative of UAMS-1. To generate protease-deficient *rot* and *sigB* mutants, we used the pKOR1 mutagenesis system [39] to knock out each gene in this same protease-deficient strain. Specifically, upstream and downstream primer pairs (Table 3) were used to amplify fragments upstream and downstream of *rot* and *sigB*, respectively. For each set of PCR products, the upstream and downstream amplification products were digested with BamHI and ligated prior to PCR amplification using the furthest upstream and furthest downstream primers used to generate the original PCR products for each gene. For each set of PCR products, upstream and downstream fragments for each gene were digested with BamHI and ligated prior to PCR amplification using the external upstream and downstream primers used to generate the original PCR products (Table 3). The resulting PCR products were then cloned into the temperature-sensitive plasmid pKOR1 using Gateway^TM^ LR Clonase II (Thermo Fisher Scientific, Cat. # 11,791,020) according to the manufacturer’s recommendations. Plasmid transfer and allelic exchange was then carried out as described above.

All strains were maintained at −80°C in tryptic soy broth (TSB) containing 25% (v/v) glycerol. For each experiment, the appropriate strains were recovered from cold storage by plating on TSA with appropriate antibiotic selection. Antibiotics were used in the following concentrations: erythromycin, 5 or 10 μg ml^−1^; chloramphenicol, 10 μg ml^−1^; tetracycline, 5 μg ml^−1^; kanamycin, 50 μg ml^−1^; neomycin, 50 μg ml^−1^; spectinomycin, 1 mg ml^−1^. Kanamycin and neomycin were always used together to avoid spontaneously resistant strains.

### Murine bacteremia model

Bacterial strains were recovered from cold storage at −80°C by plating on TSA with appropriate antibiotic selection. Multiple colonies recovered from these primary plates were inoculated into TSB with the appropriate antibiotic selection and grown to stationary phase (16 hrs) at 37°C. Each strain was then inoculated into fresh TSB without antibiotics to a standardized optical density (OD_560_) of 0.05. Cultures were then grown to an OD_560_ of 1.0. Bacterial cells were harvested by centrifugation, washed in sterile phosphate-buffered saline (PBS), and resuspended in an equal volume of PBS containing 10% DMSO and 5% bovine serum albumin (BSA). The number of colony-forming units (cfu) in this suspension was then confirmed by plate count on TSA. Aliquots of this suspension were then stored at −80°C.

For each *in vivo* experiment, bacteria were recovered from these standardized, frozen aliquots, washed with PBS, and standardized in PBS without additives to a cell density of 5 × 10^8^ cfu per ml. 5–8 week-old female NIH-Swiss mice were infected via tail vein injection of 100 µl (5 × 10^7^ cfu) of this standardized suspension. Mice were evaluated six times daily and the spleen, kidney, and heart harvested from any mice found dead or judged to require compassionate euthanasia; otherwise, tissues were harvested at 7 days post-infection. Organs were removed aseptically and homogenized using the Bullet Blender 5 Gold Bead Homogenizer (Next Advance). Serial dilutions were plated on TSA without antibiotic selection and the number of cfu per organ determined following overnight incubation at 37°C. All *in vivo* experiments were done using 10 mice per experimental group.

### Preparation and analysis of stationary-phase conditioned medium (CM)

Bacterial strains were recovered from frozen stock cultures and grown overnight (16 hr). Cultures of each strain were then standardized to an optical density of 560 nm (OD560) of 10.0. CM was then prepared from each standardized culture by removing bacterial cells by centrifugation followed by filter sterilization.

Total protease activity was assessed using CM from stationary-phase bacterial cultures. Assays were done using the Protease Fluorescent Detection Kit, which uses casein as the protease substrate (Sigma Chemical Co., Cat. #PF0100), and the EnzChek Gelatinase/Collagenase Assay Kit (Thermo Fisher Scientific, Cat. #E12055). Protease assays included two biological replicates with six experimental replicates of each.

Exoprotein profiles were examined by SDS-PAGE using 4–12% gradient Novex Bis-Tris Plus gels (Life Technologies, Cat. #NW04125BOX). Proteins were visualized by staining with SimplyBlue^TM^ SafeStain (Life Technologies, Cat. #LC6060) and imaged using Bio-Rad ChemiDocMP Imaging System (Bio-Rad Laboratories, Inc.). Western blots for extracellular protein A (eSpa), NucA, and NucB were done using commercially available antibodies (Sigma and Toxin Technologies, respectively) as previously described [36,40,41]. Western blots included two biological replicates with two experimental replicates of each.

### Assessment of biofilm formation

Biofilm formation was assessed *in vitro* using a microtiter plate assay [23]. Assays were done in 96-well microtiter plates after coating overnight at 4°C with 100 μl of 20% human plasma in carbonate/bicarbonate buffer. Bacterial cultures grown overnight in TSB supplemented with 3% sodium chloride and 0.5% glucose (biofilm medium, BFM) were standardized to an OD_560_ of 0.05 in fresh BFM. Plasma was aspirated and each well of the microtiter plate inoculated with 200 μl of this standardized culture. Plates were then incubated statically overnight at 37°C. Wells were then washed, fixed, and stained with crystal violet as previously described [23]. After staining, wells were washed three times with 250 μl PBS and left to dry overnight. The following day, the stain was eluted with 100 μl 100% EtOH for 10 min, and the eluent diluted in a new 96-well plate to achieve values within the linear range of the assay. Absorbance measurements were taken at 595 nm with a FLUOstar Omega microplate reader (BMG Labtech) and adjusted for the dilution factor as necessary. Biofilm assays included two biological replicates with six experimental replicates of each.

### Capsule immunoblotting

Quantitative analysis of capsular polysaccharide production was done by western dot blot as previously described [42]. Briefly, overnight cultures were grown for 16 hours and standardized to an OD_660_ of 5.0. Bacterial cells were harvested by centrifugation, washed with sterile PBS, and resuspended in 50 µl of sterile PBS. Cell suspensions were sequentially incubated with 0.5 µl of lysostaphin (10 mg/ml) and DNase I (2 units/µl), both at 30 min intervals at 37°C. This was followed by the addition of 0.5 µl of proteinase K (10 mg/ml) and a further incubation at 50°C for 30 min. This step was repeated before incubating at 75°C for an additional 15 min and clarification by centrifugation. Samples were serially diluted and 1.5 μl of each dilution spotted directly to a dry nitrocellulose membrane. The amount of capsule polysaccharide was determined using an anti-type 8 capsule antibody and the same immunoblotting protocol used for PIA detection as previously described [24].

### PIA immunoblotting

Production of the polysaccharide intercellular adhesion (PIA) was also assessed as previously described [24]. Briefly, cultures were grown overnight (16 hrs) in BFM and standardized to an OD_660_ of 5.0. Bacterial cells were harvested by centrifugation and resuspended in 60 μl 0.5 M EDTA. Cell suspensions were boiled for 5 min followed by centrifugation at 14,000 x g for 2 minutes. 20 μl of Tris-buffered saline (20 mM Tris-HCl, 150 mM NaCl [pH 7.4]) was added to each sample before adding 1 μl proteinase K (10 mg/ml) and incubating for 10 minutes at 37°C and then 10 minutes at 70°C. For analysis, 2 μl of each sample was spotted directly onto a dry nitrocellulose membrane and PIA detected using an anti-PIA antibody as previously described [24].

### Statistical analysis

All *in vitro* experiments were done with at least two biological replicates, each of which included at least three experimental replicates. In some cases, the results observed with the parent strain were averaged and set to a value of 1.0. The results observed with each isogenic mutant were then plotted and analyzed relative to this value. In other experiments, results were plotted as raw values, with these values used for statistical analysis. Statistical analysis was done using ANOVA methods with Dunnett’s procedure to compare the results observed with each mutant to that observed with the isogenic parent strain or by paired t-test as appropriate. For analysis of *in vivo* survival studies, log-rank (Mantell-Cox) tests were used to assess the statistical significance of the results observed with each mutant relative to the parent strain or between isogenic mutants and their protease-deficient derivatives. For analysis of bacterial burdens, ANOVA methods with Dunnett’s procedure were used to compare the results observed with each mutant to that observed with the isogenic parent strain. For this analysis, cfu data was log_10_-transformed prior to analysis and p values calculated using permutation methods. Statistical analyses were performed using GraphPad Prism 5.0 (La Jolla). P-values ≤ 0.05 were considered statistically significant.

### Ethics statement

Experiments involving animals were reviewed and approved by the Institutional Animal Care and Use Committee of the University of Arkansas for Medical Sciences and performed according to NIH guidelines, the Animal Welfare Act, and US Federal law.

## Results

### The impact of different regulatory loci on virulence in UAMS-1

**Figure 1.**
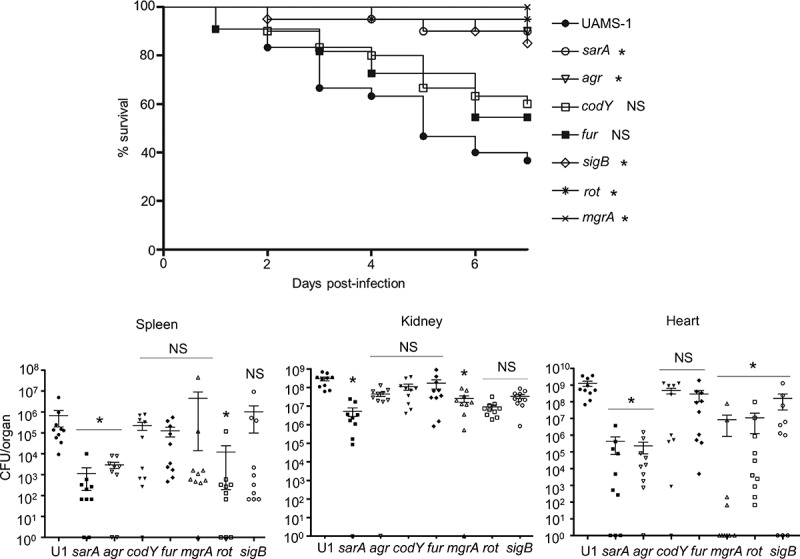
Relative virulence of *S. aureus* regulatory mutants in acute sepsis. Top: Kaplan-Meier survival curves are shown for the USA200 strain UAMS-1 and the indicated isogenic mutants. Numbers in parenthesis indicate p values for each mutant by comparison to the results observed with LAC. NS = not significant. **Bottom**: The number of colony-forming units (CFU) in the spleen, kidney, and heart are shown by scatter plot. Asterisks above each plot statistically significant values by comparison to the parent strain UAMS-1 (U1). Error bars indicate standard error of the mean of log_10_ transformed values. Asterisk indicates statistical significance relative to the parent strain. NS = not significant

To better understand how regulatory mutations that affect biofilm formation also impact the pathogenesis of diverse clinical isolates of *S. aureus* in a murine sepsis model, we generated isogenic mutants of the USA200 strain UAMS-1 in *sarA, agr, codY, fur, sigB, rot*, and *mgrA*. This strain was chosen because we carried out the same study previously with the USA300 strain LAC, and while LAC and UAMS-1 are both clinical isolates, they are very different by comparison to each other as detailed above. The potential importance of such differences is illustrated by the results we report. Specifically, in our previous report focusing on the impact of these mutations on the virulence of LAC in the same sepsis model, we found that mutation of *codY, sarA*, and *sigB* resulted in a comparable decrease in virulence, while mutation of *mgrA* and *rot* increased virulence by comparison to the isogenic parent strain [26]. In contrast, mutation of all of the loci examined in UAMS-1 other than *fur* and *codY* was found to attenuate virulence in UAMS-1 as assessed by lethality in our sepsis model (Figure 1). These results were generally consistent with bacterial burdens observed in the spleen, kidney and heart. Specifically, statistically significant reductions in bacterial burden in the spleen were observed with UAMS-1 *agr, sarA*, and *rot* mutants, while in the heart they were observed with *agr, mgrA, rot, sarA* and *sigB* mutants (Figure 1). Bacterial burdens in the kidney were less discriminatory in that only mutation of *mgrA* and *sarA* resulted in statistically significant reduction. Thus, the only mutant that exhibited reduced lethality and reduced bacterial burdens in all three organs examined was the UAMS-1 *sarA* mutant. Conversely, the only mutants that did not exhibit reduced bacterial burdens in any organ were the *fur* and *codY* mutants (Figure 1).

### Correlation between virulence, protease production, the accumulation of extracellular proteins, and biofilm formation

**Figure 2.**
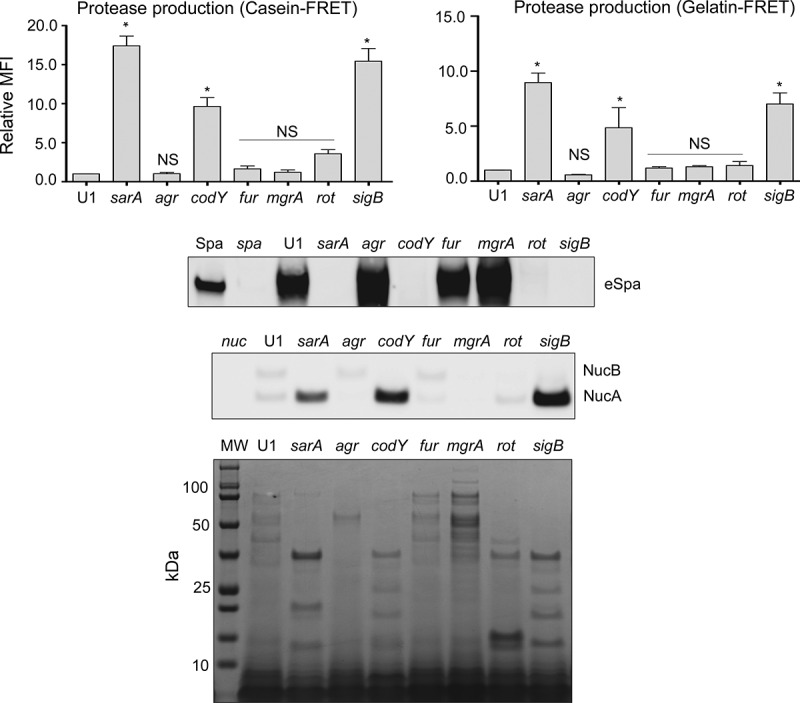
Impact of regulatory mutations on protease production in UAMS-1. Top: Total protease activity in conditioned medium (CM) was assessed with UAMS-1 (U1) and the indicated regulatory mutants using a commercially available casein-based FRET assay (left) or a gelatin-based FRET assay (right). Results obtained with U1 with each protease substrate were averaged and set to a value of 1.0. Results observed with all other strains are shown relative to this value. Bar charts are representative of results from at least two biological replicates for each of which included three experimental replicates. Results for each mutant are reported as mean fluorescence values (MFIs) ± the standard error of the means. Asterisks indicates statistical significance relative to the parent strain. NS = not significant. **Middle**: Abundance of extracellular protein A (eSpa) and the alternative forms of Nuc1 (NucA and NucB) as assessed by western blot. **Bottom**: SDS-PAGE profiles of CM from UAMS-1 and the indicated isogenic regulatory mutants

In our previous report focusing on LAC regulatory mutants in our sepsis model, we found a potentially important correlation between increased protease production and reduced virulence [26]. To examine this correlation in UAMS-1, we assessed protease production in each of these regulatory mutants. As in LAC, protease production was significantly increased in *codY, sigB*, and *sarA* mutants (Figure 2). The greatest increase was observed with the UAMS-1 *sarA* mutant followed by the *sigB* and *codY* mutants, respectively. These same relative relationships were also observed in the corresponding LAC mutants [26]. Mutation of *rot* resulted in a modest increase in protease production (Figure 2). We did not observe any increase in protease production with UAMS-1 *agr, fur*, or *mgrA* mutants.

To assess the phenotypic impact of these changes in protease production, we examined the impact of these mutations on extracellular protein profiles. With the exception of the *fur* mutant, essentially all of these mutants were associated with a visible change in exoprotein profiles, but the most striking changes were a decrease in the abundance of high molecular weight extracellular proteins, and a corresponding increase in the abundance of lower molecular weight proteins, in *sarA, codY, rot* and *sigB* mutants (Figure 2). In contrast, there was an apparent increase in the abundance of high molecular weight proteins in the UAMS-1 *mgrA* mutant. These results must be interpreted with caution in that all of the regulatory loci targeted in these studies are known to impact the production of exoproteins, but the correlation between these results and overall protease activity in *sarA, codY, sigB* and even *rot* mutants is suggestive of a cause-and-effect relationship between increased protease production and decreased accumulation of full-length high molecular weight exoproteins.

*S. aureus* produces protein A (Spa) in both surface-associated and extracellular forms [43,44], and in a previous report, we demonstrated that both forms are present in reduced amounts in CM from LAC *codY, sarA* and *sigB* mutants [26]. In a subsequent report we demonstrated that this was also true in a UAMS-1 *sarA* mutant and that the abundance of extracellular protein A (eSpa) was fully restored in both LAC and UAMS-1 *sarA* mutants by limiting the ability of these mutants to produce extracellular proteases [41]. Based on this, we examined the abundance of eSpa in other UAMS-1 regulatory mutants. The results demonstrated that eSpa is absent in CM prepared from UAMS-1 *sarA, codY, rot* and *sigB* mutants (Figure 2). With the exception of *codY*, all of these regulatory loci have been implicated in expression from the *spa* promoter and the production of Spa [45,46], but this nevertheless suggests that the increased production of extracellular proteases is likely to play some role in defining the abundance of eSpa in these mutants in both LAC and UAMS-1.

**Figure 3.**
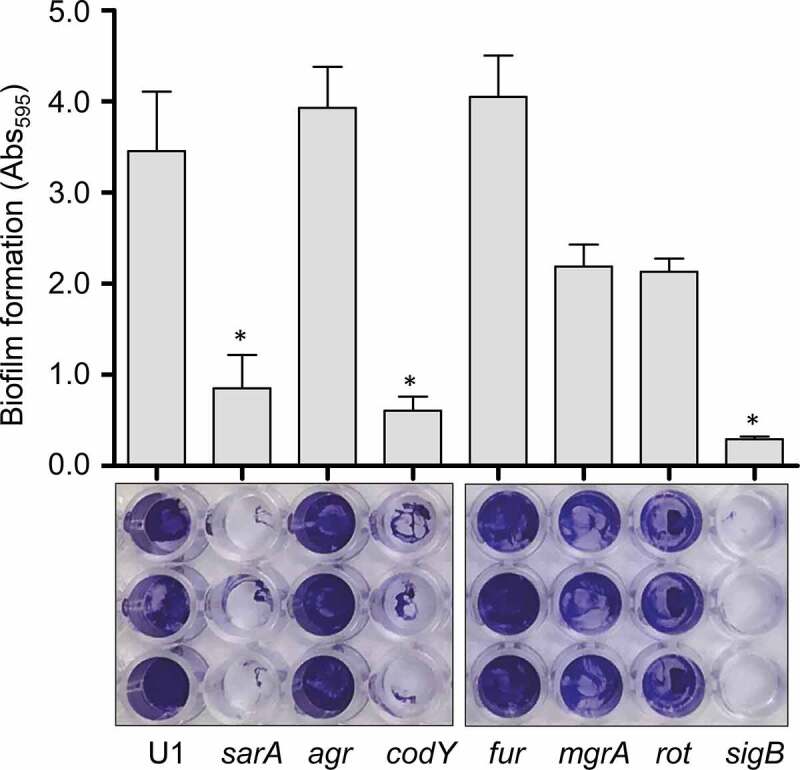
Impact of regulatory mutations on biofilm formation in UAMS-1. The relative capacity of UAMS-1 (U1) and its isogenic regulatory mutants to form a biofilm was assessed using a microtiter plate assay. Bottom image is a representative assay. Upper bar graph indicates cumulative absorbance values obtained from three biological replicates. Error bars indicate standard error of the mean. Asterisk indicates statistical significance (p ≤ 0.05) relative to the isogenic parent strain

**Figure 4.**
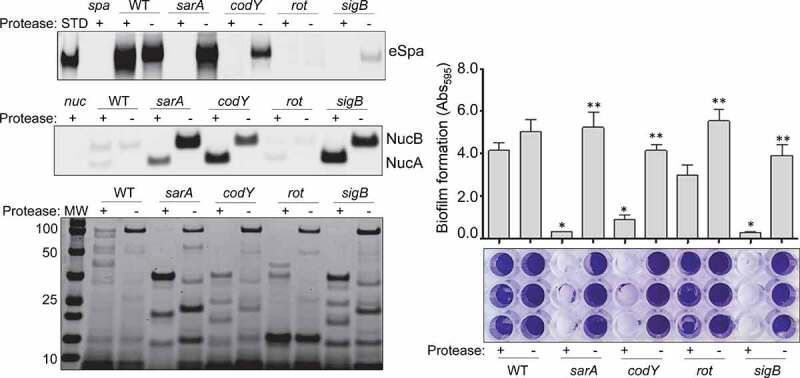
Impact of extracellular proteases in UAMS-1. Top left: Abundance of extracellular protein A (eSpa) was assessed by western blot using conditioned medium (CM) and an anti-Spa monoclonal antibody. Strains included were UAMS-1 (U1, a protease-deficient derivative of U1 (p), isogenic *sarA* (SA), *codY* (c), *rot* (r), and *sigB* (SB) mutants, and protease-deficient derivatives of each regulatory mutant (SAP, CP, and SBP, respectively). Purified protein A (Spa) and CM from a protein A mutant (*spa*) were included as positive and negative controls, respectively. **Middle left**: Abundance of extracellular nuclease (Nuc1) was assessed by western blot in the same strains. CM from a nuclease mutant (*nuc*) was included as a negative control. **Bottom left**: SDS-PAGE analysis of CM from the same *S. aureus* strains. MW: Molecular size markers. **Right**: The relative capacity of the same strains to form a biofilm was assessed using a microtiter plate assay. Graph indicates cumulative absorbance values obtained from three biological replicates. Error bars indicate standard error of the mean. Single asterisk indicates statistical significance relative to the isogenic parent strain. Double asterisks indicate significance relative to the isogenic regulatory mutant

Similarly, *S. aureus* produces an extracellular nuclease (Nuc1) as a larger protein (NucB) that is proteolytically cleaved to a smaller active form designated NucA [47]. As assessed by western blot, both forms were detected in CM from UAMS-1, but only the NucA form was detected in CM from UAMS-1 *sarA, codY, rot*, and *sigB* mutants (Figure 2). Neither form of nuclease was detected in CM from the *mgrA* mutant, and protease production was not increased in this mutant, thus making it likely that the absence of NucA and NucB in the UAMS-1 *mgrA* mutant is due to a lack of production rather than protease-mediated degradation. Biofilm formation was also reduced in all of these mutants, although in the case of *rot* the reduction was not statistically significant as assessed using our methods (Figure 3). *S. aureus* has the capacity to produce at least 10 extracellular proteases (aureolysin, ScpA, SspA, SspB and the six *spl*-encoded proteases) [48]. Eliminating the ability to produce these proteases enhanced biofilm formation in all four mutants including the *rot* mutant. It also restored the accumulation of high molecular weight proteins, at least partially restored the accumulation of eSpa, and resulted in a decrease in NucB degradation as evidenced by the reduced abundance of NucA (Figure 4).

### Impact of protease production in specific UAMS-1 and LAC regulatory mutants

**Figure 5.**
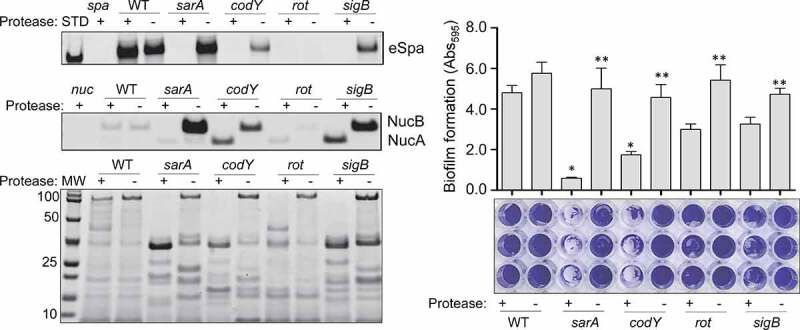
Impact of extracellular proteases in LAC. Top left: Abundance of extracellular protein A (eSpa) was assessed by western blot using conditioned medium (CM) and an anti-Spa monoclonal antibody. Strains included were LAC, a protease-deficient derivative of LAC (p), isogenic *sarA* (SA), *codY* (c), *rot* (r), and *sigB* (SB) mutants, and protease-deficient derivatives of each regulatory mutant (SAP, CP, RP and SBP, respectively). Purified protein A (Spa) and CM from a protein A mutant (*spa*) were included as positive and negative controls, respectively. **Middle left**: Abundance of extracellular nuclease (Nuc1) was assessed by western blot in the same strains. CM from a nuclease mutant (*nuc*) was included as a negative control. **Bottom left**: SDS-PAGE analysis of CM from the same *S. aureus* strains. MW: Molecular size markers. **Right**: The relative capacity of the same strains to form a biofilm was assessed using a microtiter plate assay. Graph indicates cumulative absorbance values obtained from three biological replicates. Error bars indicate standard error of the mean. Single asterisk indicates statistical significance relative to the isogenic parent strain. Double asterisks indicate significance relative to the isogenic regulatory mutant

In our previous report, we demonstrated a correlation between reduced virulence and increased protease production in LAC *codY, sarA*, and *sigB* mutants [26]. Based on the results described above for our UAMS-1 mutants, we also directly examined the impact of eliminating protease production in LAC *codY, rot, sarA*, and *sigB* mutants. All of the phenotypes discussed above were also evident in the LAC mutants, although with respect to biofilm formation, the reductions we observed with *rot* and *sigB* mutants did not reach statistical significance (Figure 5). Nevertheless, eliminating protease production in LAC also enhanced biofilm formation, restored the accumulation of eSpa, limited the degradation of NucB, and generally restored overall exoprotein profiles in all four LAC mutants (Figure 5).

### Impact of regulatory mutations and the role of increased protease production on virulence in LAC and UAMS-1

**Figure 6.**
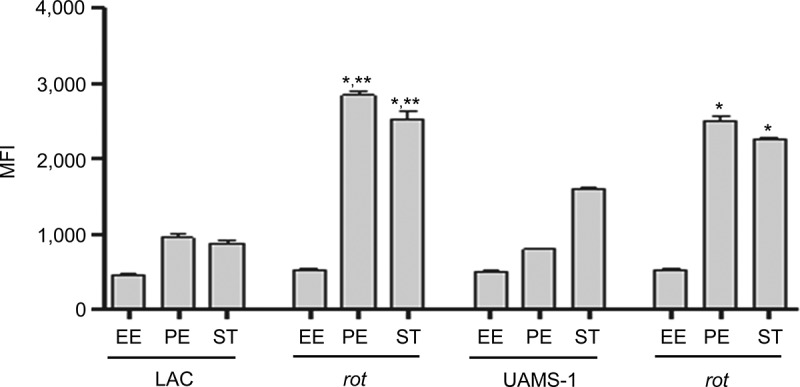
Impact of *rot* on protease production in LAC and UAMS-**1**. Total protease activity was assessed with LAC, UAMS-1, and their isogenic *rot* mutants using conditioned medium (CM) harvested at the early exponential (EE), post-exponential (PE) and stationary growth phases (ST). Protease activity was assessed using a commercially available gelatin-based FRET assay. Bar charts are representative of results from at least two biological replicates for each of which included three experimental replicates. Results are reported as mean fluorescence values (MFIs) ± the standard error of the means. Asterisk indicates statistical significance by comparison to the isogenic parent strain at the same growth phase. Double asterisks indicate statistical significance by comparison to the corresponding UAMS-1 *rot* mutant at the same growth phase

**Figure 7.**
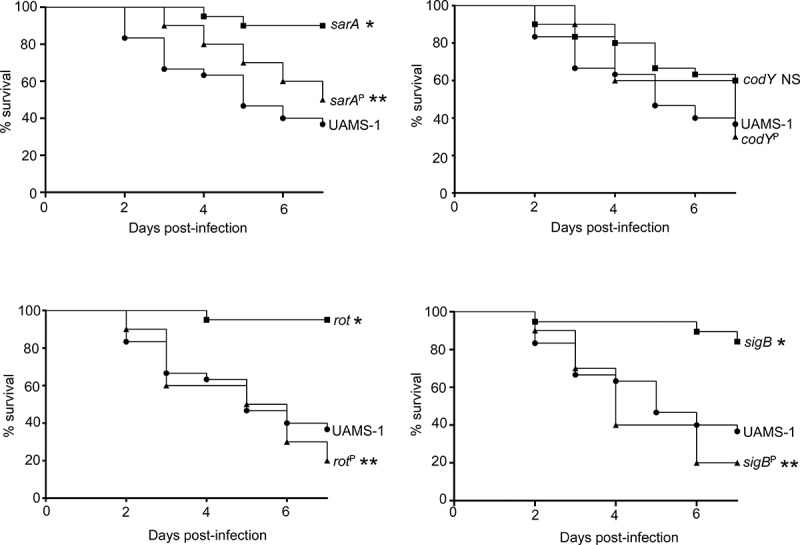
Impact of protease production on virulence in UAMS-1. A murine sepsis model was used to assess the relative virulence of UAMS-1, its *sarA, codY, rot*, and *sigB* mutants, and derivatives of each of these mutants unable to produce any extracellular protease (*sarA*^P^, *codY*^P^, *rot*^P^, and *sigB*^P^, respectively). Numbers indicate P values relative to the UAMS-1 parent strain. NS indicates no statistical significance by comparison to UAMS-1

**Figure 8.**
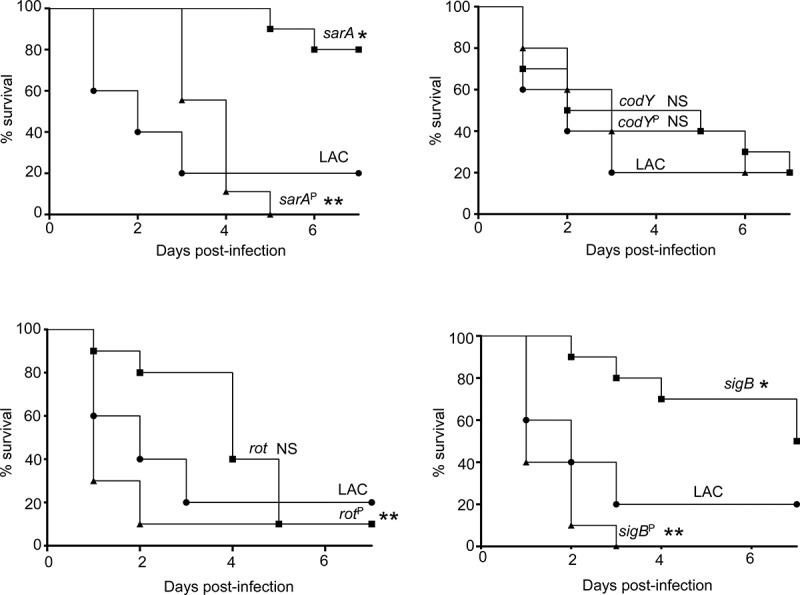
Impact of protease production on virulence in LAC. A murine sepsis model was used to assess the relative virulence of LAC, its *sarA, codY, rot*, and *sigB* mutants, and derivatives of each of these mutants unable to produce any extracellular protease (*sarA*^P^, *codY*^P^, *rot*^P^, and *sigB*^P^, respectively). Numbers indicate P values relative to the LAC parent strain. NS indicates no statistical significance by comparison to LAC

The results demonstrating a comparable impact of increased protease production in LAC and UAMS-1 *rot* mutants were somewhat surprising given that mutation of *rot* had relatively little impact in this regard by comparison to *codY, sigB* and *sarA* mutants in both UAMS-1 (Figure 2) and LAC [26]. Based on this, we examined the impact of mutating *rot* on protease production using CM from early-exponential, post-exponential, and stationary-phase cultures of both LAC and UAMS-1 (OD_560_ of 1.5, 4.0, and 10.0, respectively). The results confirmed that protease production is significantly increased in both strains during the post-exponential and stationary growth phases (Figure 6). Thus, these results suggest that even relatively modest increases in protease production can have biologically significant effects. To examine this further, we compared LAC and UAMS-1 to their isogenic regulatory mutants and protease-deficient regulatory mutants in our murine sepsis model. The results of these studies confirmed that mutation of *rot, sarA*, and *sigB* attenuated the virulence of UAMS-1, while mutation of *codY* had no significant effect (Figure 7). They also confirmed that eliminating the ability to produce any of the 10 primary *S. aureus* extracellular proteases restored the virulence of *rot, sarA*, and *sigB* mutants, but had little impact on the virulence of the *codY* mutant. Although mutation of *rot* did not have a significant impact on virulence in LAC, it did in UAMS-1, and in both strains eliminating protease production enhanced virulence while mutation of *codY* had relatively little effect (Figures 7 and 8).

### Impact of regulatory mutations on PIA and capsule production in UAMS-1

**Figure 9.**
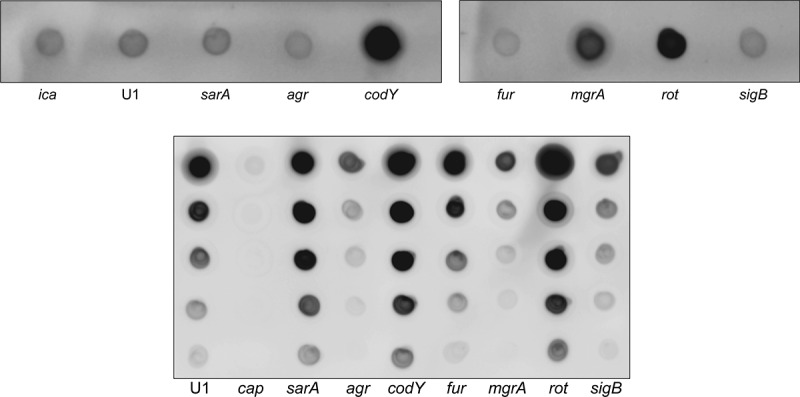
Impact of regulatory mutations on PIA and capsule production. Top: PIA production was assessed by dot blot using antibodies specific for PIA as previously described [41]. An *ica* mutant was included as a negative control. **Bottom**: Capsule production was assessed by dot blot after serial dilution using antibodies specific for type-8 capsular polysaccharide as previously described [42]. A *cap* mutant was included as a negative control

With respect to the impact of increased protease production on virulence, the biggest discrepancy we observed was associated with *codY*, mutation of which resulted in a significant increase in the production of extracellular proteases but had relatively little impact on virulence. With respect to LAC, this was in contrast to our previous report indicating that mutation of *codY* attenuated virulence in our sepsis model almost to the same degree as mutation of *sigB* and *sarA* [26]. We have no explanation for this other than the inherent variability of *in vivo* experiments. Nevertheless, the discrepancy between protease production and virulence in a *codY* mutants suggests an important contribution of protease-independent phenotypes. One possibility is production of the polysaccharide intercellular adhesin (PIA) and/or capsular polysaccharides. Interestingly, the production of both of these was dramatically enhanced in a UAMS-1 *codY* mutant (Figure 9). We have also confirmed that PIA production is also increased in a LAC *codY* mutant, albeit to a lesser extent than in a UAMS-1 *codY* mutant [37]. Although we did not examine capsule production in a LAC *codY* mutant, this nevertheless suggests that the increased production of one or both of these polysaccharides could limit the attenuation of *codY* mutants despite their increased production of extracellular proteases.

## Discussion

There is an increasing need for alternative strategies to combat *S. aureus* infections, and increasing interest in anti-virulence approaches that target key regulatory loci [18,19]. The two best-studied regulatory loci in this regard are the accessory gene regulator (*agr*) and the staphylococcal accessory regulatory (*sarA*), and putative inhibitors of the function of these loci have been described [20–22]. However, given that *S. aureus* causes diverse forms of infection, it is imperative to consider regulatory loci as therapeutic targets in the context of these diverse forms of infection. For instance, mutation of *agr* generally reduces toxin production, suggesting that inhibitors of *agr* may be helpful in the context of acute, toxin-mediated infections. However, mutation of *agr* also enhances biofilm formation [23,25]. Thus, such inhibitors could have the adverse consequence of increasing the likelihood of chronic, biofilm-associated infections. Indeed, it is not uncommon to isolate *agr* mutants from chronically infected patients [49], and there is one report that examined sequential isolates from the same patient and concluded that mutations that limit the regulatory functions of *agr* can be directly correlated with phenotypic adaptations that favor chronic infections including osteomyelitis [50].

This issue is further complicated by the complexity of *S. aureus* regulatory circuits and the genetic and phenotypic diversity among clinical isolates. For instance, one report used comparative genomics tools to construct a model of the regulatory network of *S. aureus* and six related staphylococcal species. This network included 46 transcription factors with more than 1,900 binding sites, 2,800 target genes, and approximately 320 regulatory interactions between these transcription factors, and the results suggested that only 20% of the interactions in *S. aureus* regulatory circuits are conserved among all *S. aureus* strains [51].

We have begun to address these issues by making direct comparisons between the relative impact of different regulatory loci in diverse animal models and in clinical isolates that are demonstrably different from each other. Given our specific interest in orthopedic infection and the important role of biofilms in these infections, we initially focused on the impact of *S. aureus* regulatory loci on biofilm formation. This led us to place a primary focus on *sarA*, mutation of which limits biofilm formation in both LAC and UAMS-1 to a greater degree than any other regulatory locus we have examined [24].

We also demonstrated that mutation of *sarA* limits virulence in murine models of implant-associated infection, sepsis, and osteomyelitis, and that this is due in all cases to the increased production of extracellular proteases [24,26,36,37,41,52–57]. In the USA300, methicillin-resistant strain LAC, we found that mutation of *codY* and *sigB* attenuated virulence in a murine sepsis model to a degree comparable to that observed in an isogenic *sarA* mutant [26]. One phenotype that all of these mutants had in common was the increased production of extracellular proteases. We had previously evaluated the impact of these additional regulatory loci on biofilm formation [24,52]. However, our sepsis studies were limited to LAC, thus leaving open the question whether a similar degree of attenuation would be observed with these regulatory mutants in other clinical isolates of *S. aureus*. To address this, we generated mutations in the USA200, methicillin-sensitive strain UAMS-1, which was isolated originally from the bone of an osteomyelitis patient during surgical debridement [28].

As in our previous study with LAC, mutations were generated in UAMS-1 in *agr, codY, fur, mgrA, rot, sarA* and *sigB*. With respect to the impact of these mutations on virulence in our sepsis model, we did observe some differences between LAC and UAMS-1. Specifically, in UAMS-1, mutation of *agr, mgrA*, and *rot* limited virulence, while mutation of *codY* did not, and this was not the case with the corresponding LAC mutants [26]. Thus, only mutation of *sarA* and *sigB* resulted in reduced virulence in both LAC and UAMS-1.

Defining the mechanistic reasons for such differences is extremely complicated by the complexity and highly interactive nature of *S. aureus* regulatory circuits, but the results observed with the UAMS-1 *codY* mutant were particularly surprising in that mutation of *codY* resulted in a comparable increase in protease activity in both strains and the hypothesis that increased protease production attenuates virulence owing to the decreased accumulation of *S. aureus* virulence factors. However, mutation of *codY* has been shown to result in increased expression of *agr* in *S. aureus* [58,59], and while it would therefore be anticipated that mutation of *codY* would result in increased protease production as was observed in the UAMS-1 *codY* mutant, it would also be anticipated that it would result in increased expression of *agr*. To the extent that *agr* significantly enhances the production of multiple *S. aureus* virulence factors [1], this could provide a counter-balance to the increased production of extracellular proteases, particularly in a strain like UAMS-1 that expresses *agr* at relatively low levels by comparison to a strain like LAC [37]. Similarly, mutation of *sigB* results in increased expression of *agr*, but also decreased expression of *sarA* [60]. With respect to the impact of *sigB* on *sarA*, there are reports to the contrary [61], but we found that the amount of SarA present in whole cell lysates is reduced in LAC and UAMS-1 *sigB* mutants by comparison to the isogenic parent strains (data not shown). While mutation of *sigB* did not abolish the production of SarA, this suggests that the increased production of extracellular proteases in *sigB* mutants could be mediated by the impact of *sigB* on production of SarA as a repressor of protease production even despite its impact on the expression of *agr*. Addressing this issue will require additional experiments that are beyond the scope of this report, but this nevertheless emphasizes the complexity of *S. aureus* regulatory circuits, the need to assess the impact of critical elements within this circuit in diverse clinical isolates, and the potential importance of the balance between the production of *S. aureus* virulence factors and their protease-mediated degradation.

Mutation of *rot* also reduced the virulence of UAMS-1 in our sepsis model but enhanced virulence in LAC [26]. As with *codY* and *sigB*, this difference is difficult to explain in that *rot* also impacts expression of both *agr* and *sarA*. Specifically, RNAIII, the primary effector molecule of the *agr* system, blocks translation of *rot* mRNA, and *rot* expression is repressed by both SarA and SigB [1]. However, Rot also influences the production of *S. aureus* virulence factors directly, and this includes extracellular proteases. For instance, a recent report demonstrated that mutation of *rot* increases protease production via direct binding to the promoters of the corresponding genes and that this increase is sufficient to limit the capacity of LAC to form a biofilm [62]. There is also a recent report confirming that mutation of *rot* results in increased expression of the gene encoding aureolysin (*aur*) and the *sspABC* operon and increased protease activity as assessed by gelatin-based zymography [63]. We found that, by comparison to mutation of *codY, sigB* and *sarA*, mutation of *rot* had only a modest impact on protease production, and we were not able to demonstrate a significant decrease in biofilm formation in a LAC *rot* mutant [24].

Nevertheless, this is consistent with our observation that mutation of *rot* in both LAC and UAMS-1 led to a decreased accumulation of high molecular weight proteins and increased accumulation of lower molecular weight proteins. Although *rot* is known to serve multiple regulatory roles that impact exoprotein production [64], we believe this shift from high to lower molecular weight proteins is likely to reflect degradation of at least some *S. aureus* extracellular proteins resulting from an overall increase in protease activity. Further support for this comes from the absence of eSpa in CM from a UAMS-1 *rot* mutant. However, Rot has been shown to bind the *spa* promoter and stimulate transcription [65], thus suggesting that the absence of Rot would result in reduced production of protein A irrespective of its impact on protease production. To the extent that protein A has been shown to promote biofilm formation [66], its absence in a *rot* mutant might be expected to limit biofilm formation as previously indicated [62],

Taken together, these results emphasize the complexity and interactive nature of *S. aureus* regulatory circuits, and by extension the need to address the impact of different regulatory loci in divergent clinical isolates in the context of phenotypes of potential clinical relevance and, more importantly, virulence in relevant animal models. One of these phenotypes that has been found to be increasingly important in recent years is protease production. Specifically, while extracellular proteases play important roles in tissue invasion, the acquisition of nutrients, and subversion of host defenses, it has also been shown that eliminating protease production increases the virulence of LAC owing to the increased abundance of important virulence factors [67]. Conversely, mutation of *sarA* limits virulence in murine models of sepsis and osteomyelitis owing to the increased production of these proteases and corresponding decrease in the availability of these same virulence factors [36,54,55,57,68].

Based on this, we focused in this report on directly assessing the impact of each regulatory mutation on protease production in UAMS-1 and its impact on these phenotypes, including virulence in our sepsis model. As with LAC, mutation of *rot, codY, sarA* and *sigB* all resulted in a significant increase in overall protease production. Also as in LAC, mutation of *sarA* had the greatest effect followed by mutation of *sigB, codY*, and *rot*, respectively. Biofilm formation was limited to a significant degree in UAMS-1 *codY, sarA*, and *sigB* mutants, which with the exception of *codY*, was consistent with the results previously generated using these same mutants in both LAC and UAMS-1 [24]. Mutation of *codY* in UAMS-1 did result in a significant increase in protease production, and in this report we confirm that this contributes significantly to the reduced capacity of a UAMS-1 *codY* mutant to form a biofilm. However, the biofilm phenotype we report is in direct contrast with what has previously been reported, which indicated that mutation of *codY* in UAMS-1 had little impact on biofilm formation [24,69]. One possible explanation for this disparity is that the biofilm assays were sometimes compromised by what appeared to be intracellular aggregation of the *codY* mutant, which resulted in a tendency for the entire aggregate to be removed during the washing steps (data not shown). This is consistent with the dramatic increase in the production of PIA in a *codY* mutant [24,37]. Thus, the increased production of extracellular proteases in *codY* mutants may limit the abundance of proteins required for the initial adherence phase of biofilm formation but simultaneously result in increased intercellular aggregation. However, this does not preclude the possibility that the increased production of extracellular proteases in a UAMS-1 *codY* mutant impacts relevant phenotypes of *S. aureus*, and in fact we demonstrated that this is the case as evidenced by the reduced accumulation of high molecular weight proteins and changes in the abundance and/or form of eSpa and Nuc1.

This was also true with *rot, sarA* and *sigB* mutants, and in all three cases we confirmed that increased protease production makes a significant contribution to their reduced virulence in our sepsis model. Thus, we believe the results we report provide strong support for the hypothesis that a key but largely overlooked component of *S. aureus* regulatory circuits is the ability to limit the production of extracellular proteases such that they serve their intended purposes without compromising the abundance of critical *S. aureus* virulence factors. Moreover, they demonstrate that this is the case in diverse clinical isolates of *S. aureus* as represented by the USA300, methicillin-resistant strain LAC and the USA200, methicillin-sensitive strain UAMS.
